# Nomogram for predicting postoperative pulmonary infection in elderly patients undergoing major orthopedic surgery

**DOI:** 10.3389/fmed.2025.1537697

**Published:** 2025-05-16

**Authors:** Yuhan Liu, Yunping Fan, Xuping Yang, Haibin Gan, Xiaohua Li, Yanrong Luo, Qianyun Pang, Tingjun Yang

**Affiliations:** ^1^Department of Anesthesiology, Shizhu Tujia Autonomous County People's Hospital, Chongqing, China; ^2^Department of Anesthesiology, Chongqing University Cancer Hospital, Chongqing, China

**Keywords:** orthopedic surgery, postoperative infection, pulmonary, aged, nomogram

## Abstract

**Objective:**

The incidence of pulmonary infection following major orthopedic surgery in the elderly is high, significantly affecting prognosis. Identifying high-risk factors and stratifying patient risk more effectively is an urgent problem that needs to be addressed. This study aims to develop a nomogram for predicting postoperative pulmonary infection (PPI) in elderly patients undergoing major orthopedic surgery.

**Methods:**

Data from preoperative variables, surgical procedures, and anesthesia factors of 814 elderly patients who underwent major orthopedic surgery between January 2020 and October 2023 were retrospectively collected to develop a nomogram. The primary outcome was PPI. Stata 16 and R 4.1.2 software were used for statistical analysis.

**Results:**

Multivariate logistic regression revealed that gender (OR = 2.336, 95% CI1.135–4.807, *p* = 0.021), preoperative pulmonary disease (OR = 6.042, 95% CI 2.849–12.814, *p* = 0.000), preoperative sedation and analgesia (OR = 0.159, 95% CI 0.037–0.689, *p* = 0.014), intraoperative infusion volume ≥ 1,200 mL (OR = 2.530, 95% CI 1.166–5.489, *p* = 0.019) were identified as independent risk factors for PPI in elderly orthopedic patients. The risk factors in the nomogram included ASA, gender, preoperative pulmonary disease, cognitive impairment, and non-preoperative sedation and analgesia, and intraoperative infusion. Area under the curve (AUC) of the nomogram was 0.834, the slope was 1.000, and the net benefit of the decision curve analysis (DCA) curve was 0.01–0.60.

**Conclusion:**

Researchers have developed and validated a predictive nomogram for PPI in elderly patients undergoing major orthopedic surgery, identifying 6 key variables, which can be used to predict PPI of aged patients undergoing major orthopedic surgery and identify high risk groups.

## 1 Introduction

Among countries, China has one of the fastest aging populations (1). The risks and challenges of surgical anesthesia for aged patients are substantial, especially for aged patients undergoing major orthopedic surgery. These patients are more susceptible to postoperative pulmonary infections (PPIs) because of preoperative frailty, numerous underlying systemic conditions, and restricted perioperative activity. The incidence of PPI in major orthopedic surgery in the elderly ranges from 3.5% to 14.4% (2, 3). Postoperative pulmonary complications have a significant impact on perioperative morbidity and mortality, prolong postoperative hospital stays, and greatly increase hospital costs (4).

Numerous studies have investigated the high-risk factors for PPI in aged patients, leading to the development of predictive models to identify susceptible individuals early. However, most studies to date have concentrated on hip replacement surgery (5, 6), leaving uncertainly regarding the applicability of these PPI prediction models to major orthopedic surgeries. Preoperative pain, stress and sleep disturbances can increase postoperative complications, which may affect PPI (7). And preoperative pain control can alleviate postoperative pain and improve sleep disorders (8). However, most studies only include patient factors and surgical-related factors, neglecting preoperative pain control and anesthesia-related perioperative factors. Numerous studies have shown that anesthesia and analgesia methods are associated with PPI (9, 10). Therefore, this study retrospectively analyzed the perioperative clinical data, including patient demographics, surgical procedures and anesthesia-related perioperative factors, and specifically preoperative sedative and analgesia practices, among aged patients undergoing major orthopedic surgery in our hospital. The aim was to develop a risk prediction model for PPI in aged patients undergoing major orthopedic surgery, to aid medical staff in early identification of high-risk patients and to offer theoretical support and a simple practical tool.

## 2 Materials and methods

### 2.1 Study design

This study was approved by the Institutional Ethics Committee of Shizhu Tujia Autonomous County People’s Hospital, China (Scientific Ethics Review No. 18 in 2022). Due to the retrospective nature of the study and the anonymity of the data, informed consent was not necessary. To ensure anonymity, names and admission and surgical dates were removed during data extraction.

### 2.2 Patients and sample size

According to Harrell guidelines and reference study from Riley et al. (11), the number of outcome variables should be at least 10–20 times the number of variables. Based on prior studies, approximately 15% of elderly patients undergoing major orthopedic surgery developed PPI. In our study design, we aim to incorporate 12 predictive factors to enhance the accuracy of our prognosis models. To achieve this, we determine that a minimum of 800 patients was necessary to ensure the robustness and statistical significance of our findings. Accounting for a 20% dropout rate, we aimed to enroll at least 960 patients in the study.

Patients who underwent major orthopedic surgery at Shizhu Tujia Autonomous County People’s Hospital spanning from January 2020 to October 2023 were enrolled. The inclusion criteria were as follows: (1) patients who underwent major orthopedic surgery (surgical time >60 min) in our hospital; and (2) age ≥ 65 years old. The exclusion criteria were as follows: (1) patients who underwent orthopedic surgery under local anesthesia; (2) superficial and minimally invasive surgeries such as mass excisions, tendon repairs, and arthroscopies; (3) severe trauma with brain trauma, unconsciousness, pulmonary contusion, hemothorax and pneumothorax, rib fractures, etc.; and (4) incomplete clinical medical records.

### 2.3 Data collection

In our hospital, the electronic medical record system and surgical anesthesia system were queried using special medical terms such as “fracture” or “arthroplasty” or “replacement” or “vertebra” or “spine” to identified relevant patient records and surgical-anesthesia procedure. Six researchers used a retrospective study method to extract data from patients’ electronic medical records, test records, examination reports, and nursing records. All raw data were collected using self-designed case report form. Two investigators reviewed the data to ensure its accuracy and completeness. Any disagreement was settled by discussion among all researchers.

Observation indicators included (1) patient factors: age, sex, New York Heart Association (NYHA) classification, smoking history, combined pulmonary disease [chronic obstructive pulmonary disease (COPD), asthma, pulmonary infection, tuberculosis], cerebrovascular disease (history of stroke, brain atrophy), cognitive impairment (diagnosed dementia or Montreal Cognitive Assessment Scale < 26 points), diabetes, coronary heart disease, hypertension, preoperative arterial partial pressure of oxygen, pulmonary function, hemoglobin, leukocyte, neutrophil-to-lymphocyte ratio (NLR), albumin, and creatinine; and (2) surgery procedure and anesthesia-related perioperative factors: preoperative sedation and analgesia, anesthesia method, surgical duration, blood loss (ml), fluid volume (ml), blood transfusion, postoperative pain (defined as a VAS score > 3 points within 48 h after operation), postoperative analgesia method, and intensive care unit (ICU) stay.

### 2.4 Outcomes

The primary outcome was the incidence of pulmonary infection within 7 days of surgery, and the secondary outcomes were length of postoperative hospital stay (LOS) and in-hospital mortality. PPI refers to the diagnosis of postoperative pneumonia defined in the systematic review conducted by Abbott et al. (12): chest X-ray (without underlying cardiopulmonary disease) with at least 1 of the following imaging features: new or progressive persistent pulmonary infiltrates, consolidations, or cavities; at least 1 of the following symptoms: unexplained fever (>38°C), leukocyte (<4 × 10^9^/L) or leukocyte (>12 × 10^9^/L) and, for elderly individuals over 70 years old, disturbance of consciousness with no other explanation; at least 2 reasons: new onset cough, aggravated sputum, change in sputum color, increased respiratory secretions, increased need for sputum suction; new onset or aggravated cough, dyspnoea, shortness of breath; pulmonary rales or bronchial breath sounds; and worsening of gas exchange (hypoxemia, increased oxygen demand, and increased need for mechanical ventilation).

### 2.5 Missing data

Data cleaning was performed before statistical analyses, and variables with more than 10% missing values were not included. For continuous variable values that were missing (within 10%), the mean or median was used instead according to whether the data follows a normal distribution or not.

### 2.6 Statistical analysis

Categorical variables are represented by the number of cases, and were analyzed using the chi-square test. Continuous variables are expressed as medians and interquartile ranges (IQRs), and were analyzed using the t test or rank-sum test. Univariate logistic regression analysis was performed to screen the risk factors, and the variables with *p* < 0.1 were included in the multivariate logistic analysis with forward-backward stepwise method based on the AIC criteria to screen the variables that were ultimately included in the nomogram model. Then, a nomogram was constructed based on the results of the multiple regression.

The area under the curve (AUC) and the calibration curve were used to evaluate the discrimination and accuracy of the nomogram model. Decision curve analysis (DCA) was used to assess the range of clinical validity of the model. All analyses were conducted using Stata 16 (Stata Corp) and R Software 4.1.2 (R Foundation for statistical computing) (Supplementary material 1). For all analyses, *p* < 0.05 was considered statistically significant.

## 3 Results

In this study, 10 patients were excluded due to severe trauma, and 4 patients were excluded due to incomplete data. Ultimately, 814 patients were enrolled in the analysis.

### 3.1 General characteristic of the included patients

The incidence of PPI in this study was 4.7% (38 cases), and 1 patient died due to a PPI. The median LOS in the PPI group was 14 days, and the median LOS in the non-PPI group was 10 days; the difference between the 2 groups was significant (*p* = 0.003). Preoperative arterial partial pressure of oxygen, pulmonary function and albumin were excluded from the analysis because the missing data exceeded 10%. Between the two groups of patients, there were significant differences in gender, ASA classification, combined pulmonary disease and cognitive impairment, preoperative sedation and analgesia, operation time, blood loss, intraoperative fluid infusion, blood transfusion, and postoperative ICU stay (*p* < 0.05, Table 1).

**Table 1:** Characteristics of the patients with or without PPI.

Variable	PPI (*n* = 38)	Non-PPI (*n* = 776)	Z value	*p* value
Age (year)	73.5 (69, 80)	71 (67, 78)	−1.429	0.153
Gender (male/female)	14/24	461/315		0.006
ASA (I/II/III/IV)	0/22/15/1	4/595/175/2		0.008
NYHA (I/II/III/IV)	7/29/2/0	227/512/37/0		0.354
Surgical site (Proximal/distal)	15/23	298/475		0.909
Emergency (yes/no)	1/37	21/755		0.978
Smoke (yes/no)	5/33	99/677		0.942
Comorbidity				
Pulmonary disease (yes/no)	23/19	133/836		0.000
Diabetes (yes/no)	3/35	74/702		0.736
Cardiac artery disease (yes/no)	3/39	76/893		0.869
Hypertension (yes/no)	15/23	314/462		0.903
Cerebrovascular disease (yes/no)	8/30	97/679		0.125
Cognitive impairment (yes/no)	4/34	11/765		0.000
Blood test				
Hemoglobin (g/L)	114 (102, 125)	117 (104,130)	1.282	0.200
Creatinine (umol/L)	63 (56, 72)	59 (51,70)	−1.208	0.227
Leukocyte (*10^9^/L)	6.75 (4.67, 8.73)	6.66 (5.40,8.30)	0.388	0.698
NLR	3.85 (2.46, 8.39)	4.12 (2.78,6.66)	0.055	0.956
Preoperative sedation and analgesia (yes/no)	2/36	211/565		0.003
Anesthesia method (GA or GA + LA vs. SEA or LA)	16/22	300/476		0.670
Surgical time (min)	125 (95, 175)	115 (85, 149)	−2.194	0.028
Bleeding (ml)	150 (100, 300)	100 (50, 200)	−2.870	0.004
Intraoperative infusion (ml)	1,200 (1,000, 2,000)	1,000 (500, 1,300)	−3.708	0.000
Transfusion (yes/no)	15/23	173/603		0.014
Postoperative pain (yes/no)	11/27	254/522		0.627
Analgesia method (systematic/LA)	33/5	565/211		0.056
ICU stay (yes/no)	5/33	26/750		0.002
LOS (d)	14 (10, 15)	10 (7, 14)	−2.941	0.003

Data are median [lower quartile to upper quartile] and No. of cases. Z value for continuous variables. PPI, postoperative pulmonary infection; ASA, American society of Anesthesiologists; NYHA, New York Heart Association; WBC, white blood cell; NLR, neutrophil-to-lymphocyte ratio; GA, general anesthesia; SEA, spinal or epidural anesthesia; LA, local anesthesia; ICU, intensive care unit; LOS, postoperative length of hospital stay.

### 3.2 Univariate and multivariate logistic regression results

Univariate analysis showed that ASA classification, gender, combined pulmonary disease and cognitive impairment, preoperative sedation and analgesia, intraoperative fluid infusion, and blood transfusion were associated with PPI (*p* < 0.05). The multivariate analysis results showed that gender (OR = 2.336, 95% CI 1.135, 4.807, *p* = 0.021), preoperative pulmonary disease (OR = 6.042, 95% CI 2.849, 12.814, *p* = 0.000), intraoperative fluid infusion volume ≥1,200 mL (OR = 2.530, 95% CI 1.166, 5.489, *p* = 0.019) were independent risk factors and preoperative sedation and analgesia (OR = 0.159, and 95% CI 0.037, 0.689, *p* = 0.014) was independent protective factor for PPI in elderly patients undergoing major orthopedic surgery (Table 2).

**Table 2:** Univariate and multivariate logistic analyses.

Variable	Univariate logistic analysis			Multivariate logistic analysis		
	OR	95%CI	*p*-value	OR	95%CI	*p*-value
Age (year) (≥75 vs. < 75)	1.541	0.732, 3.243	0.255			
Gender (male vs female)	2.509	1.278, 4.925	0.008	2.336	1.135, 4.807	0.021
ASA (III/IV vs. I/II)	2.461	1.265, 4.788	0.008	1.515	0.681, 3.369	0.308
NYHA (III/IV vs. I/II)	1.110	0.257, 4.786	0.889			
Smoke (yes vs. no)	1.036	0.395, 2.717	0.942			
Pulmonary disease (yes vs. no)	7.028	3.599, 13.724	0.000	6.042	2.849, 12.814	0.000
Diabetes (yes vs. no)	0.813	0.244, 2.708	0.736			
Cardiac artery disease (yes vs. no)	0.954	0.285, 3.187	0.938			
Hypertension (yes vs. no)	0.960	0.493, 1.868	0.903			
Cerebrovascular disease (yes vs. no)	1.867	0.832, 4.189	0.130			
Cognitive impairment (yes vs. no)	8.182	2.477, 27.024	0.001	2.814	0.645, 12.285	0.169
Surgical site (Proximal vs. distal)	1.040	0.534, 2.024	0.909			
Leukocyte (≥10*10^9^ vs. < 10*10^9^)	1.141	0.434, 2.996	0.790			
NLR (≥7.0 vs. < 7.0)	1.157	0.552, 2.428	0.699			
Creatinine (≥96 umol/L vs. < 96 umol/L)	2.285	0.771, 6.769	0.136			
Preoperative sedation and analgesia (yes vs no)	0.149	0.036, 0.623	0.009	0.159	0.037, 0.689	0.014
Anesthesia method (GA or GA + LA vs. SEA or LA)	1.154	0.596, 2.233	0.671			
Surgical time (≥120 min vs. < 120 min)	1.735	0.903, 3.332	0.098	1.000	0.995, 1.005	0.954
Bleeding (≥200 mL vs. < 200 mL)	1.542	0.802, 2.963	0.194			
Fluid infusion (≥1,200 mL vs <1,200 mL)	2.874	1.475, 5.599	0.002	2.530	1.166, 5.489	0.019
Transfusion (yes vs. no)	2.273	1.161, 4.452	0.017	1.182	0.544, 2.570	0.673
Postoperative pain (yes vs. no)	0.837	0.409, 1.715	0.627			
Analgesia method (systematic vs. LA)	1.154	0.596, 2.233	0.671			
ICU stay (yes vs. no)	4.371	1.578, 12.104	0.005			
LOS (d)	2.803	1.309, 6.000	0.008			

OR, odds rate; CI, confidence interval; ASA, American society of Anesthesiologists; NYHA, New York Heart Association; NLR, neutrophil-to-lymphocyte ratio; GA, general anesthesia; SEA, spinal or epidural anesthesia; LA, local anesthesia; ICU, intensive care unit; LOS, postoperative length of hospital stays.

### 3.3 Development of a nomogram for PPI

**Figure 1:**
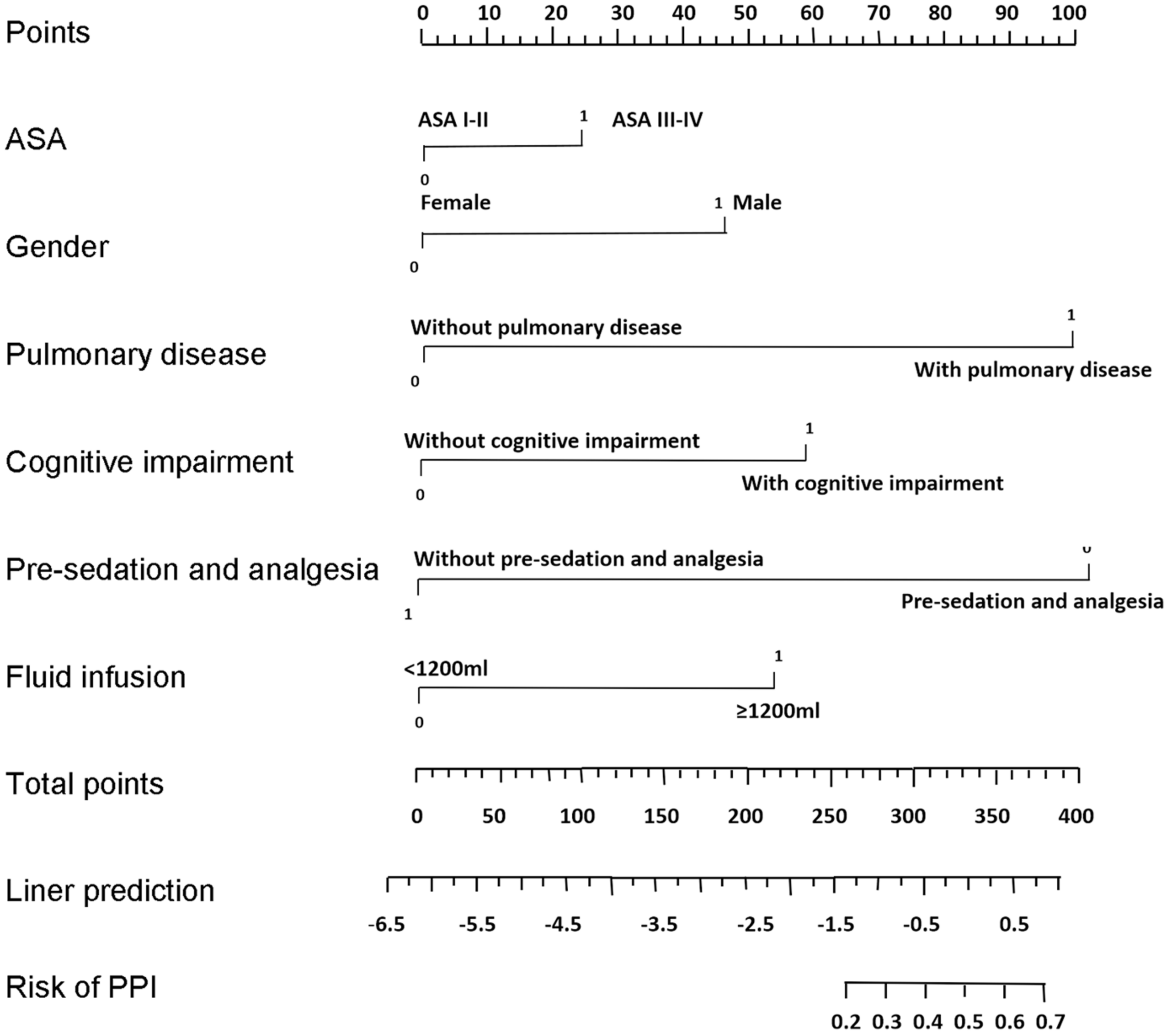
Nomogram for predicting the risk of PPI in elderly patients undergoing major orthopedic surgery. To use the nomogram, a vertical line is drawn up to the top point row to assign points for each variable. Then, the total number of points is calculated, and a vertical line is drawn downward from the total point row to obtain the probability of PPI.

Eight variables, i.e., ASA, gender, pulmonary disease, cognitive impairment, preoperative sedation, intraoperative fluid infusion, surgical time, and blood transfusion were included in stepwise regression. Finally, a nomogram was constructed incorporating 6 risk factors: ASA, gender, pulmonary disease, cognitive impairment, absence of preoperative sedation and analgesia, and intraoperative fluid infusion (Figure 1). The scores for these indicators in the nomogram were 23.8, 45.5, 98, 56.1, 100, and 52.5, respectively.

### 3.4 Validation of the nomogram for PPI

**Figure 2:**
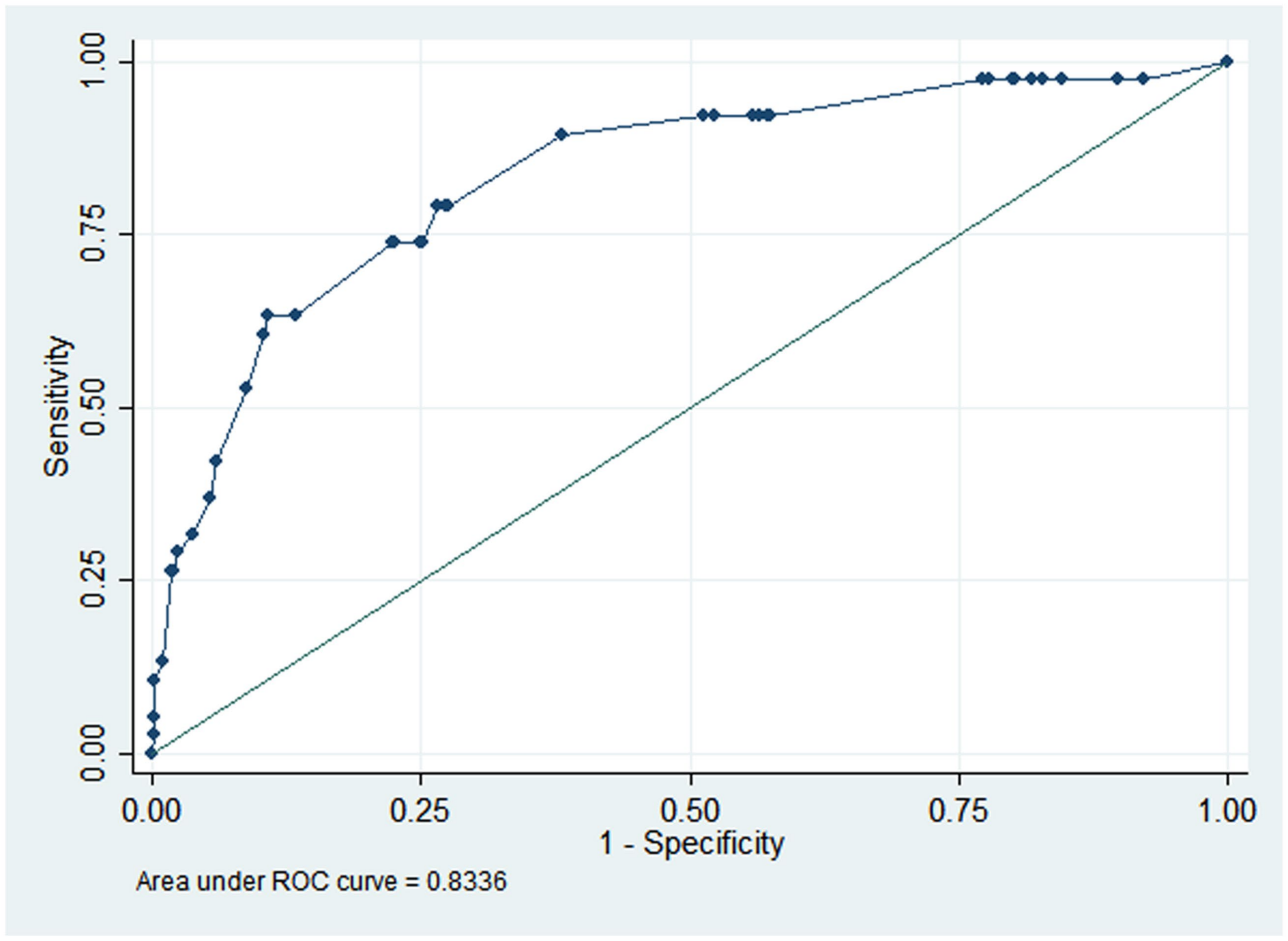
AUC of nomogram for predicting PPI in elderly patients undergoing major orthopedic surgery. The AUC was 0.834, indicating high discrimination. AUC, area under receiver-operating characteristic curve; PPI, postoperative pulmonary infection.

**Figure 3:**
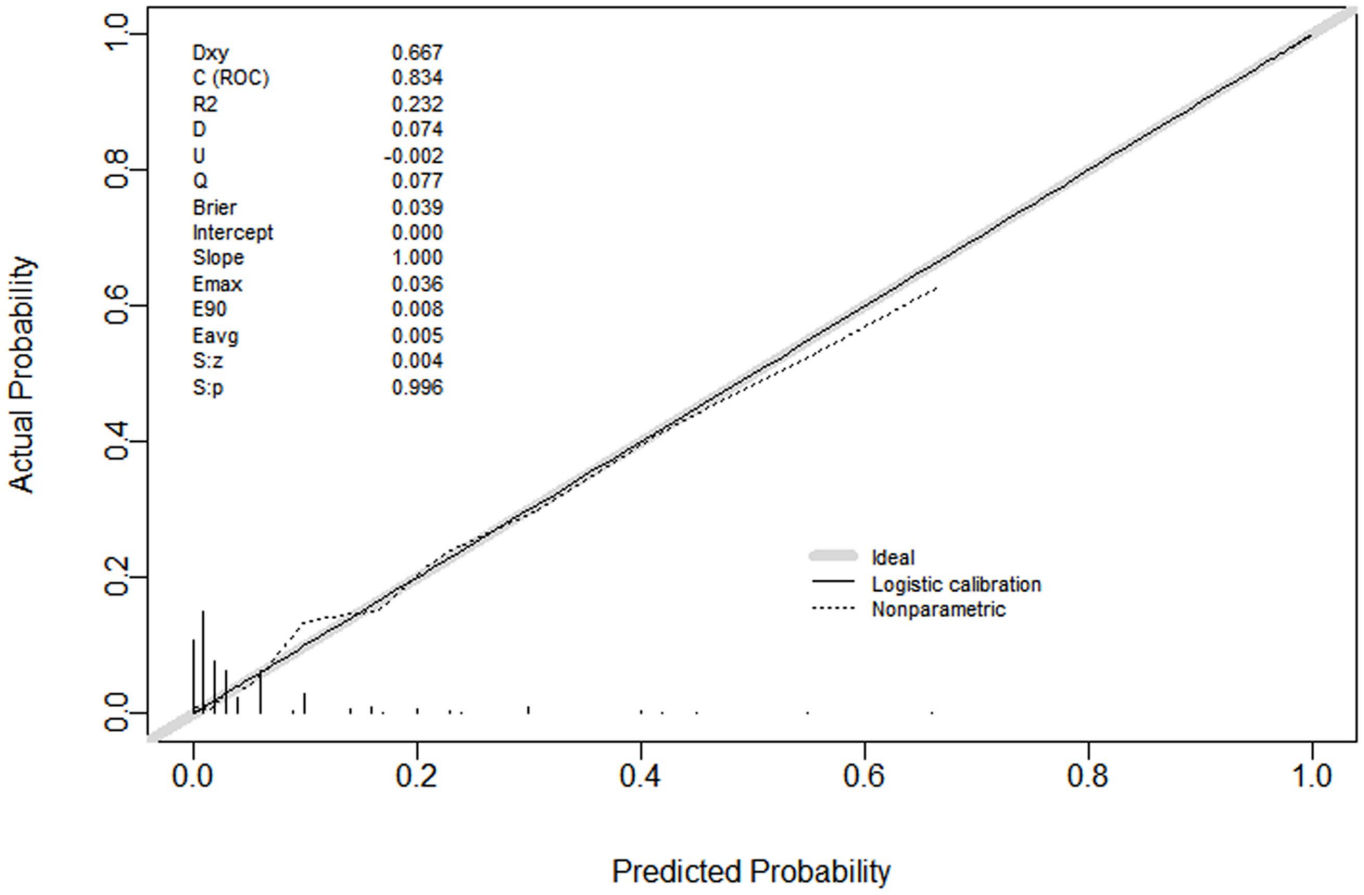
Calibration curve of nomogram for predicting PPI in elderly patients undergoing major orthopedic surgery. The slope was 1.0, the R^2^ was 0.232. The calibration curve showed good concordance between predicted probability and actual probability. PPI, postoperative pulmonary infection.

**Figure 4:**
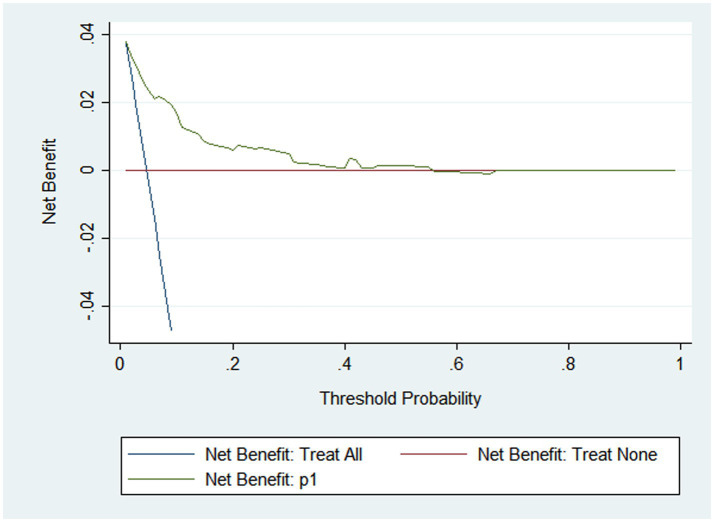
DCA of nomogram for predicting PPI in elderly patients undergoing major orthopedic surgery. DCA, decision curve analysis; PPI, postoperative pulmonary infection.

The AUC was 0.834, indicating that the model exhibited strong discriminatory ability (Figure 2). A calibration curve was drawn, and the Hosmer–Lemeshow (HL) goodness-of-fit test was also conducted. The HL test result showed that *p* = 0.696, and the calibration curve was straight with a slope of 1.0, indicating good consistency between the predicted values and the observed results (Figure 3). The DCA indicated that, when the threshold probability was within a range of 0.01–0.60, the nomogram added more net benefit than the “treat all” or “treat none” strategies (Figure 4).

## 4 Discussion

Postoperative pulmonary infection (PPI) is a severe complication for elderly patients undergoing major orthopedic surgery, as evidenced by studies indicating that it not only prolongs the postoperative hospital stay but also increases the risk of readmission and death (13). One study revealed that nearly 25% of deaths in the first week after surgery were related to PPIs (14). In this study, the incidence of PPI was 4.7%, and only 1 patient died due to a PPI. Univariate and multivariate logistic regressions were used to analyze the effects of patient and surgical anesthesia factors on PPI in elderly orthopedic major surgery patients, and 6 variables were identified and incorporated into the nomogram for predicting PPI: ASA, gender, combined pulmonary disease, cognitive impairment, preoperative sedation and analgesia, and intraoperative fluid infusion. In addition, the AUC, calibration plot, and DCA showed satisfactory performance for the prediction model.

Many studies have shown that anemia, diabetes, number of comorbidities, ASA ≥ III and some specific laboratory biomarkers and significant clinical interventions are important risk factors for PPI (6, 15, 16). The risk factors incorporated into the prediction model of this study are largely consistent with those identified in prior research (17–19). Our results also found that preoperative sedation and analgesia was a protective factor for elderly orthopedic major surgery patients. Patients with orthopedic surgery often suffer from pain before operation, which affects exercise and sleep quality, and also increases preoperative stress and inflammation, which are closely related to the postoperative pain (7). Surgery also can initiate the pain process, evokes hyperalgesia, releases inflammatory factors, and leads to reduce immunity (20). Several clinical studies have shown that pre-emptive sedation and analgesia can decrease the level of postoperative pain, and allevate postoperative pain (6, 21, 22). The mechanism may involve preoperative pain control, which can help reduce perioperative IL-6 levels and hs-CRP levels, alleviate postoperative pain and improve sleep disorders (8, 21). Therefore, sedatives and analgesics should be used to improve the preoperative state of patients before orthopedic surgery.

ASA classification ≥ III means patients exhibit frailty and many comorbidities, resulting on a lower cardiopulmonary function compared to those classified as ASA I-II (22). Therefore, pulmonary complications are likely to occur after surgery. Male patients are mostly affected by long-term smoking and are often accompanied by COPD, asthma and pneumonia, which affect lung function (23). Anesthesia and surgery can reduce lung volumes, which is the primary physiologic mechanism that contributes to the development of atelectasis and other postoperative pulmonary complications (24). Compared with systemic opioids, epidural local anesthetics increased arterial partial pressure of oxygen and decreased the incidence of PPI and pulmonary complications (4, 7). But the results of this study showed that the mode of anesthesia had no effect on PPI. The reason might be that most high-risk patients underwent epidural anesthesia or nerve block, or other local anesthesia technique. The commonly used sedatives and analgesics in wards in our hospital are mainly dezocine, tramadol, or NSAIDs, which have minimal impact on respiration. Aspiration is a factor that will be strongly linked to PPI. In our study, the incidence of postoperative vomiting was 11.3%, but no aspiration was recorded in any of the groups.

Studies reported that preoperative cognitive impairment associated with morbidity and mortality, including pulmonary complications after surgery (25, 26). The mechanism may involve cognitive impairment due to ineffective respiratory exercise, leading to higher rates of moderate-deep residual sedation during anesthesia recovery. This can result in prolonged postoperative ventilation, an increased incidence of delirium, and subsequently, ineffective coughing and atelectasis (27, 28).

We used data from elderly orthopedic surgery patients at our hospital to validate two other models from studies by Zhang et al. (5) and Tian et al. (29) The results showed that the accuracy of these models was relatively low, with AUC value of 0.674 and 0.758 (Supplementary Figures 1, 2). The HL calibration test results showed *p* values of 0.010 and 0.065, respectively. Variations in regional practices and the technical abilities of surgeons and anesthesiologists may contribute to differences in model outcomes derived from data collected across various hospitals. Furthermore, discrepancies in regional healthcare standards and hospital levels can lead to variation in the extension and comprehensiveness of patient examinations, which may result in certain indicators no being part of routine examinations, thus making it challenging to obtain relevant data. Studies have indicated that preoperative oxygen partial pressure below 72.5% in elderly patients undergoing hip fracture surgery is associated with PPI (30). Certain studies suggest that pulmonary function, as well as preoperative and early postoperative hypoalbuminemia, are linked to PPI in major orthopedic surgeries (31, 32). Elevated CRP levels can also serve as a predictor for PPI in some research (6). However, since many patients in this study did not routinely receive arterial blood gas analysis, pulmonary function tests, albumin or CRP level measurements prior to surgery, these factors were excluded from the analysis. Nonetheless, their potential impact on PPI cannot be overlooked.

In our study, the nomogram’s Area Under the Curve (AUC) was 0.834, and the calibration curve’s slope was 1, *p* value was 0.696 from HL test, suggesting good consistency and calibration. The Decision Curve Analysis (DCA) demonstrated the clinical practicability of the prediction model, indicating that its discrimination ability for individual probabilities was satisfactory. Therefore, the prediction model with 6 simple clinical factors can assist clinicians in identifying high-risk patients with PPI before surgery.

However, this study has certain limitations. Firstly, the data were collected retrospectively, which may compromise the reliability of all information, potentially introducing bias into the results and increasing the risk of misdiagnosis and missed diagnosis. Some cases of postoperative atelectasis may also fulfill the diagnostic criteria of PPI and thus confound the results. Secondly, being a single-center study, the sample size was inadequate, and some risk factors were not included due to incomplete data, potentially undermining the robustness of the results. Thirdly, prospective external verification was not performed, and the application value of the model needs to be confirmed by further research.

In conclusion, this study developed a 6-factor nomogram prediction model for predicting PPI in elderly patients undergoing major orthopedic surgery, considering patients, surgical and anesthesia related factors. The model can help in the identification of high-risk individuals early and in the formulation of optimal anesthesia and perioperative management strategies to reduce the occurrence of PPIs. However, a larger sample size and a multicenter study are needed to confirm these conclusions.

## Data Availability

The original contributions presented in the study are included in the article/Supplementary material, further inquiries can be directed to the corresponding authors.

## Supplementary material

SUPPLEMENTARY MATERIAL 1 Codes used in Stata and R software.

SUPPLEMENTARY FIGURE 1 AUC of Zhang's research model for external validation with data from our hospital. The AUC was 0.674, indicating low accuracy.

SUPPLEMENTARY FIGURE 2 AUC of Tian's research model for external validation with data from our hospital. The AUC was 0.758, indicating medium accuracy.
