# Supplementary figures and images for: Nomogram for predicting postoperative pulmonary infection in elderly patients undergoing major orthopedic surgery
Source: Front Med (Lausanne). 2025 May 16;12:1537697. doi: 10.3389/fmed.2025.1537697 (PMC12122517; doi:10.3389/fmed.2025.1537697)

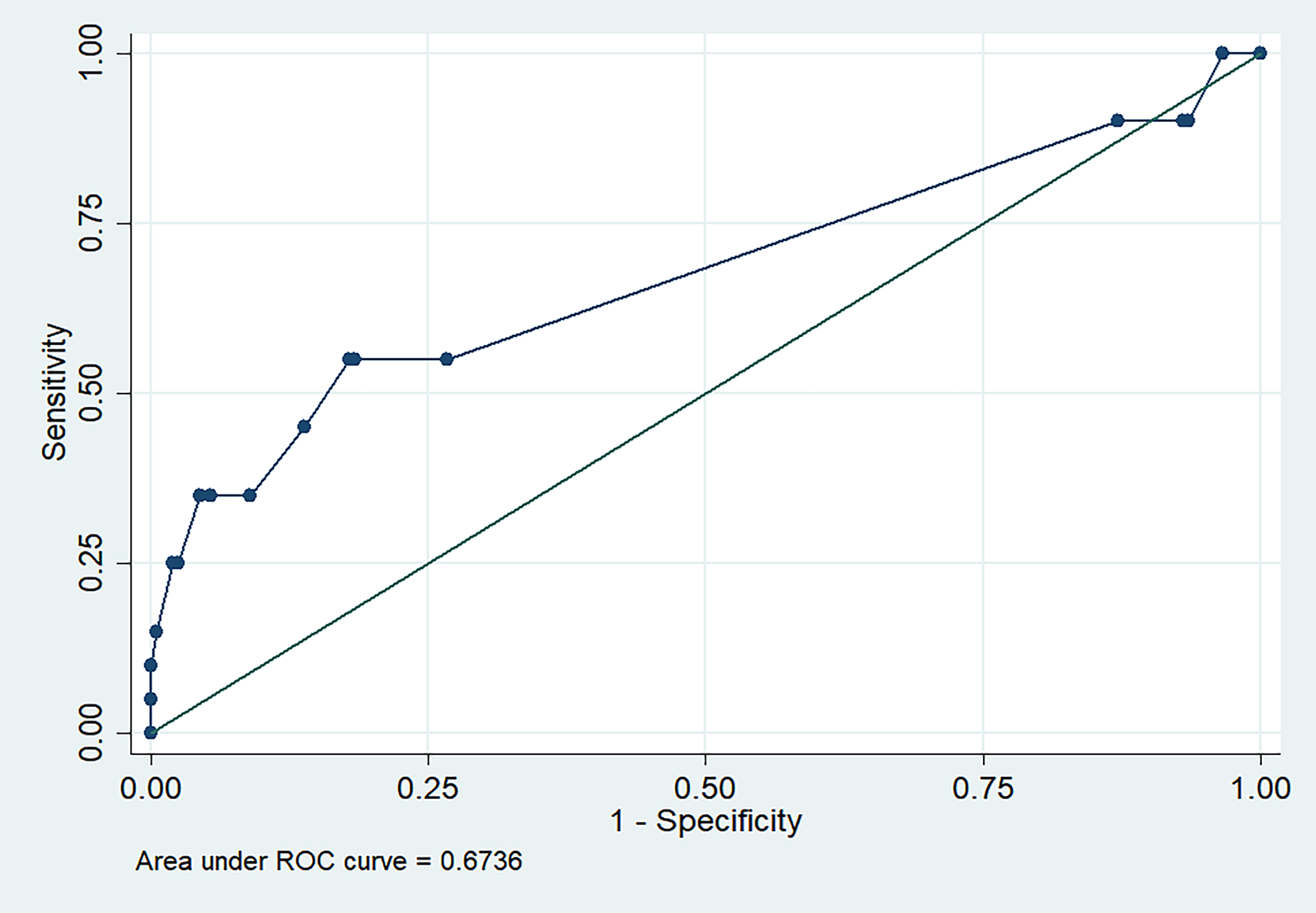

Supplement: SUPPLEMENTARY MATERIAL 1 — Codes used in Stata and R software. [file Image_1.png]

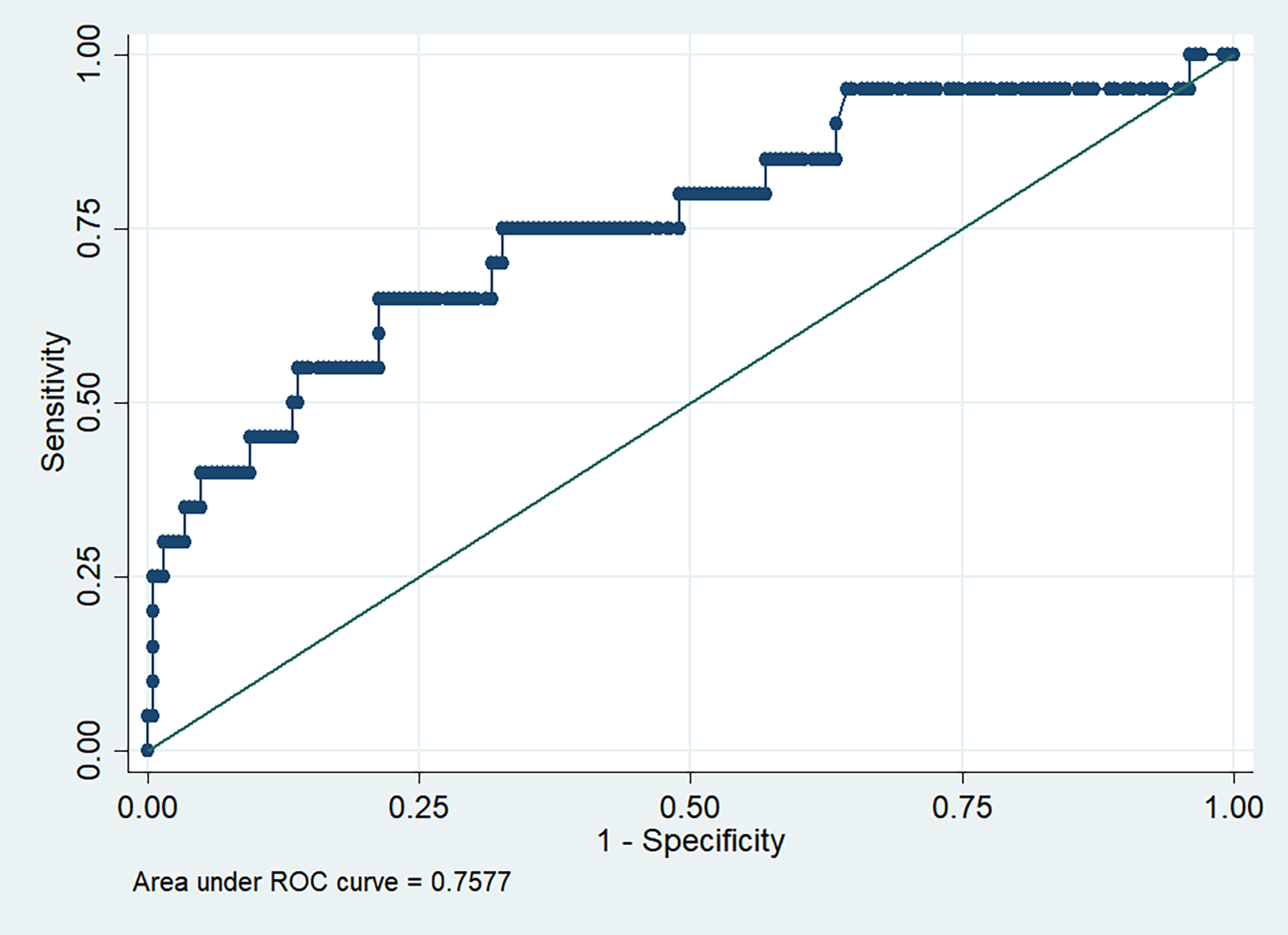

Supplement: SUPPLEMENTARY FIGURE 1 — AUC of Zhang's research model for external validation with data from our hospital. The AUC was 0.674, indicating low accuracy. [file Image_2.png]
