# Supplementary material for: Nomogram for predicting postoperative pulmonary infection in elderly patients undergoing major orthopedic surgery
Source: Front Med (Lausanne). 2025 May 16;12:1537697. doi: 10.3389/fmed.2025.1537697 (PMC12122517; doi:10.3389/fmed.2025.1537697)
Supplement: SUPPLEMENTARY FIGURE 2 — AUC of Tian's research model for external validation with data from our hospital. The AUC was 0.758, indicating medium accuracy. [file Table_1.docx]

Codes used in Stata

gen age_a=0 if age<70

replace age_a=1 if age>=70

gen age_b=0 if age<75

replace age_b=1 if age>=75

gen wbc_a=0 if wbc<10

replace wbc_a=1 if wbc>=10

gen hb_a=0 if hb>=110

replace hb_a=1 if hb<110

gen nlr_b=0 if nlr<4.0

replace nlr_b=1 if nlr>=4.0

gen cr_a=0 if cr<96

replace cr_a=1 if cr>=96

gen bleeding_a=0 if bleeding<400

replace bleeding_a=1 if bleeding>=400

gen fluid_a=0 if fluid<1500

replace fluid_a=1 if fluid>=1500

gen surgical_time_a=0 if surgical_time<150

replace surgical_time_a=1 if surgical_time>=150

gen los_a=0 if los<10

replace los_a=1 if los>=10

gen number_of_comobidity==

gen comobidity_number=0 if number_of_comobidity==0

replace comobidity_number=0 if number_of_comobidity==1

replace comobidity_number=1 if number_of_comobidity>=2

gen asa_a=0 if asa==1

replace asa_a=0 if asa==2

replace asa_a=1 if asa==3

replace asa_a=1 if asa==4

gen anesthesia_a=0 if anesthesia==2

replace anesthesia_a=0 if anesthesia==3

replace anesthesia_a=0 if anesthesia==5

replace anesthesia_a=0 if anesthesia==6

replace anesthesia_a=1 if anesthesia==1

replace anesthesia_a=1 if anesthesia==4

sktest age if ppi==0

sktest age if ppi==1

ranksum age, by(ppi)

sktest cr if ppi==0

sktest cr if ppi==1

ranksum cr, by(ppi)

sktest hb if ppi==0

sktest hb if ppi==1

ranksum hb, by(ppi)

sktest wbc if ppi==0

sktest wbc if ppi==1

ranksum wbc, by(ppi)

sktest nlr if ppi==0

sktest nlr if ppi==1

sdtest nlr,by(ppi)

ranksum nlr, by(ppi)

sktest surgical_time if ppi==0

sktest surgical_time if ppi==1

ranksum surgical_time, by(ppi)

sktest bleeding if ppi==0

sktest bleeding if ppi==1

ranksum bleeding, by(ppi)

sktest fluid if ppi==0

sktest fluid if ppi==1

ranksum fluid, by(ppi)

sktest los if ppi==0

sktest los if ppi==1

ranksum los, by(ppi)

tabstat age,stats(q)by(ppi)

tabstat cr,stats(q)by(ppi)

tabstat hb,stats(q)by(ppi)

tabstat wbc,stats(q)by(ppi)

tabstat nlr,stats(q)by(ppi)

tabstat surgical_time,stats(q)by(ppi)

tabstat bleeding,stats(q)by(ppi)

tabstat fluid,stats(q)by(ppi)

tabstat los,stats(q)by(ppi)

tabulate asa ppi, row chi

tabulate nyha ppi, row chi

tabulate gender ppi, chi2

tabulate emergency ppi, chi2

tabulate smoking ppi, chi2

tabulate alcohol ppi, chi2

tabulate cad ppi, chi2

tabulate hypertension ppi, chi2

tabulate arrythemia ppi, chi2

tabulate copulmonary_disease ppi, chi2

tabulate dm ppi, chi2

tabulate precognitive_impairment ppi, chi2

tabulate transfusion ppi,chi2

tabulate icu ppi,chi2

tabulate postoperative_pain ppi,chi2

tabulate operative_site ppi, row chi

tabulate education ppi, row chi

tabulate anesthesia ppi, row chi

tabulate sedative ppi, row chi

tabulate preemptive_analgesia ppi, row chi

tabulate postoperative_analgesia ppi, row chi

tabulate postoperative_sedation ppi, chi2

tabulate icu ppi, chi2

tabulate postoperative_analgesia ppi, row chi

tabulate transfusion ppi,chi2

#==========**Univariate logistic analyses**

logistic ppi age_a

logistic ppi gender

logistic ppi asa_a

logistic ppi nyha_a

logistic ppi smoking

logistic ppi copulmonary_disease

logistic ppi dm

logistic ppi cad

logistic ppi hypertension

logistic ppi cerebrovascular_disease

logistic ppi cognitive_disease

logistic ppi operative_site

logistic ppi wbc_a

logistic ppi nlr_b

logistic ppi cr_a

logistic ppi anesthesia

logistic ppi sedative_analgesia

logistic ppi surgical_time_a

logistic ppi fluid_a

logistic ppi transfusion

logistic ppi icu

logistic ppi postoperative_analgesia_a

logistic ppi bleeding

logistic ppi los_a

#==========**multivariate logistic analyses**

logistic ppi cognitive_disease sedative_analgesia transfusion fluid_a surgical_time_a copulmonary_disease gender asa_a transfusion

#==========**stepwise regression**

logit ppi asa_a gender cognitive_disease copulmonary_disease sedative_analgesia surgical_time fluid_a transfusion

est store model1

logit ppi asa_a gender cognitive_disease copulmonary_disease sedative_analgesia fluid_a transfusion

est store model2

logit ppi asa_a gender cognitive_disease copulmonary_disease sedative_analgesia fluid_a

est store model3

stepwise,pe(0.2):logit ppi asa_a gender cognitive_disease copulmonary_disease sedative_analgesiasurgical_time fluid_a transfusion

est store f11

stepwise,pe(0.001): logit ppi asa_a gender cognitive_disease copulmonary_disease sedative_analgesia surgical_time fluid_a transfusion

est store f21

stepwise,pr(0.02):logit ppi asa_a gender cognitive_disease copulmonary_disease sedative_analgesia surgical_time fluid_a transfusion

est store b11

stepwise,pr(0.001):logit ppi asa_a gender cognitive_disease copulmonary_disease sedative_analgesia surgical_time fluid_a transfusion

est store b21

stepwise,pe(0.2) forward:logit ppi asa_a gender cognitive_disease copulmonary_disease sedative_analgesia surgical_time fluid_a transfusion

est store fs1

stepwise,pr(0.2)pe(0.001):logit ppi asa_a gender cognitive_disease copulmonary_disease sedative_analgesia surgical_time fluid_a transfusion

est store bs1

est stats model1 model2 f11 f21 b11 b21 fs1 bs1

est stats model2 model3 f11 f21 b11 b21 fs1 bs1

lrtest model1 model2

lrtest model2 model3

model3 AIC lowest

#=============**DEVELOP A NOMOGRAM MODEL**

logit ppi asa_a gender cognitive_disease copulmonary_disease sedative_analgesia fluid_a

gen p1=1/(1+exp(-(-4.444+0.844*gender+0.441*asa_a+1.820*copulmonary_disease+1.042*cognitive_disease-1.857*sedative_analgesia+0.975*fluid_a)))

#=============**ROC**

lroc

#=============**H-Ltest**

estat gof

#=============**DCA**

dca ppi p1

Codes used in Stat R software

#============**nomogram**

setwd("D:/sz_complication/ppi")

library(readxl)

data<- read_excel("D:/sz_complication/ppi/data.xlsx")

y<-data$ppi

ASA<-data$ASA

Gender<-data$Gender

Pulmonary_disease<-data2$Pulmonary_disease

Fluid<-data$Fluid

Cognitive_disease<-data$Cognitive_disease

Sedative_analgesia<-data$Sedative_analgesia

ddist <- rms::datadist(data)

options(datadist='ddist')

f1<-rms::lrm(y~ASA+Gender+Pulmonary_disease+Cognitive_disease+Sedative_analgesia+Fluid,

data=data)

nomogram1 <- nomogram(f1,

fun=function(x)1/(1+exp(-x)),

fun.at=seq(0.2,0.8,by=0.1),

funlabel="Risk of ppi")

plot(nomogram1)

#============**Calibration curve**

library(rms)

library(foreign)

setwd("D:/sz_complication/ppi")

library(readxl)

data2<-read_excel("data.xlsx")

y<-data$ppi

p1<-data$p1

rms::val.prob(data$p1,data$ppi)

rms::val.prob(p1,y)
